# Evaluating Research Impact: The Development of a Research for Impact Tool

**DOI:** 10.3389/fpubh.2016.00160

**Published:** 2016-08-25

**Authors:** Komla Tsey, Kenny Lawson, Irina Kinchin, Roxanne Bainbridge, Janya McCalman, Felecia Watkin, Yvonne Cadet-James, Allison Rossetto

**Affiliations:** ^1^The Cairns Institute and College of Arts, Society and Education, James Cook University, Cairns, QLD, Australia; ^2^Centre for Health Research, School of Medicine, Western Sydney University, Sydney, NSW, Australia; ^3^Centre for Chronic Disease, Australian Institute of Health and Tropical Medicine, James Cook University, Cairns, QLD, Australia; ^4^School of Health and Human Services, Central Queensland University, Cairns, QLD, Australia; ^5^Australian Aboriginal and Torres Strait Islander Centre, James Cook University, Cairns, QLD, Australia; ^6^College of Business, Law and Governance, James Cook University, Townsville, QLD, Australia

**Keywords:** valuing research, “a wicked problem”, participatory learning-by-doing approaches, research users and information needs, knowledge translation, Indigenous health, Lowitja Institute

## Abstract

**Introduction:**

This paper examines the process of developing a Research for Impact Tool in the contexts of general fiscal constraint, increased competition for funding, perennial concerns about the over-researching of Aboriginal and Torres Strait Islander issues without demonstrable benefits as well as conceptual and methodological difficulties of evaluating research impact. The aim is to highlight the challenges and opportunities involved in evaluating research impact to serve as resource for potential users of the research for impact tool and others interested in assessing the impact of research.

**Materials and methods:**

A combination of literature reviews, workshops with researchers, and reflections by project team members and partners using participatory snowball techniques.

**Results:**

Assessing research impact is perceived to be difficult, akin to the so-called “wicked problem,” but not impossible. Heuristic and collaborative approach to research that takes the expectations of research users, research participants and the funders of research offers a pragmatic solution to evaluating research impact. The logic of the proposed Research for Impact Tool is based on the understanding that the value of research is to create evidence and/or products to support smarter decisions so as to improve the human condition. Research is, therefore, of limited value unless the evidence created is used to make smarter decisions for the betterment of society. A practical way of approaching research impact is, therefore, to start with the decisions confronting decision makers whether they are government policymakers, industry, professional practitioners, or households and the extent to which the research supports them to make smarter policy and practice decisions and the knock-on consequences of doing so. Embedded at each step in the impact planning and tracking process is the need for appropriate mix of expertise, capacity enhancement, and collaborative participatory learning-by-doing approaches.

**Discussion:**

The tool was developed in the context of Aboriginal and Torres Strait Islander research but the basic idea that the way to assess research impact is to start upfront with the information needs of decisions makers is equally applicable to research in other settings, both applied (horizontal) and basic (vertical) research. The tool will be further tested and evaluated with researchers over the next 2 years (2016/17). The decision by the Australian Government to include ‘industry engagement’ and ‘impact’ as additions to the Excellence in Research for Australia (ERA) quality measures from 2018 makes the Research for Impact Tool a timely development. The wider challenge is to engage with major Australian research funding agencies to ensure consistent alignment and approaches across research users, communities, and funders in evaluating impact.

## Introduction

Good decisions flow from great research

-Lowitja Institute: http://www.lowitja.org.au/lowitja-video

Aboriginal and Torres Strait Islander (hereafter, Indigenous) health literature has a long tradition of identifying and describing difficulties facing Indigenous peoples and proposing solutions. It repeatedly calls on researchers to show how their research contributes meaningfully to improving Indigenous health and wellbeing. To be truly valuable, such research must be consistent with the needs of rather than simply complying with research funding criteria. Until now, however, there has been no robust and reliable framework for evaluating the impact of Indigenous health research. This paper, a follow-up to Bainbridge et al. (1), proposes a set of steps that can guide researchers to better plan their research for impact.

The literature has often identified the “over-researching” of Indigenous Australians without demonstrable benefits (2–6). It regularly details the significant ongoing health and social disparities between Indigenous peoples and other Australians. More recently, some authors have pointed out the difficulties awaiting those who enter largely uncharted territory to assess the extent to which research contributes to improving Indigenous health. Some of the key challenges identified include defining research benefit from the point of view of research participants; focusing on societal benefits rather than more readily measurable academic benefits, such as citation frequency or number of downloads; the time lag between conducting research and using the resulting knowledge; the costs of assessing the societal impact of research; and the lack of researchers’ control over the implementation of the policy and practice changes that should flow from their research (1, 7). The assessment of research impact also suffers from the age-old problem of attribution – how to differentiate the relative contribution of research from contextual and other factors (8, 9).

This pattern is reflected in other sectors within the global research sphere. Challenges, such as aging populations, the rise in chronic diseases, social inequalities, and climate change adaptation and mitigation, are exerting pressures on healthcare and other public expenditure (10). Significant government investment in health research has, therefore, become a necessity, but this is not without expectations. Not only must researchers demonstrate the benefits in terms of preventative and curative healthcare but they must also demonstrate the social, economic, environmental, and cultural value of such research (9, 11, 12). Evidence suggests that a significant proportion of taxpayer-funded research is wasteful. According to one estimate, a staggering AU $1.4 billion per annum of global spending on health and medical research has limited benefit beyond supporting the careers of researchers (13).

Governments are not alone in calling for accountability: Indigenous peoples themselves are demanding a different kind of accountability. They call for a decolonized research agenda that positions Indigenous people at the center of Indigenous research (1, 14, 15). This suggests that the way researchers demonstrate the value of their research in the context of Indigenous health is likely to differ from the way it is conceptualized in other settings.

There is little peer-reviewed published literature on how to tackle the challenge of evaluating the impact of Indigenous health research. This is in sharp contrast to the growing body of mainstream literature on research impact tools over the past 15 years (10). One widely acknowledged best practice research impact tool is the “Payback Framework,” which is based on five areas of potential benefit impact assessment: (1) knowledge; (2) benefits to future research and research use; (3) political and administrative benefits; (4) health-sector benefits; and (5) broader economic benefits (16).

An Australian team of researchers (17) has developed a modified form of the Payback approach, called the Health Services Research Impact Framework (HSRIF). This was designed to better capture the impact of primary health care-specific research, especially in rural contexts. The authors suggested that evidence of impact should be gathered in six key areas: (1) knowledge production; (2) research targeting, capacity building; (3) informing policy and product development; (4) health and health-sector benefits; (5) broader economic benefits; and (6) research transfer. The final domain was added because of growing recognition that research transfer is relevant across all the other domains of impact assessment.

Although these tools exist, it is not always clear how they relate to or align with the major research funding agencies’ assessment criteria for prioritizing research proposals. The extent to which such tools align with assessment domains of value to Indigenous people or what Indigenous people expect of research is also unclear. Without a common understanding of what is important and what is not, researchers, funders, and Indigenous communities are likely to find themselves moving in different directions. This is a recipe for chaos, if not disaster.

There is, however, at least one example of alignment between funding body criteria and a research impact tool. This is the Australian Government’s Cooperative Research Centres (CRC) Impact Tool. Since its inception in 1992, the CRC has made it mandatory for all research grant applicants to model, in economic terms, the net impact of their proposed research (8). While this represents some progress, as with the “Payback” and other impact tools it is not clear how the CRC Tool aligns with Indigenous expectations and aspirations for research. Furthermore, the CRC Tool is specifically designed to assess research impact in broad program or theme areas, rather than at project-specific levels (8). Consequently, the wider utility of this and the other tools for evaluating Indigenous research without significant adjustments is perceived to be limited.

This paper seeks to address these challenges by proposing a research impact planning tool that ensures alignment between what Indigenous people expect of research and the criteria by which the relevant research funding agencies prioritize research. Developed by the Lowitja Institute in collaboration with the authors of this paper, the tool is intended to ensure that when evaluating the impact of Indigenous health research, communities, funders, and researchers proceed on similar rather than different courses. A brief overview of the Lowitja Institute and its efforts to improve the value of its research for Indigenous health follows, before a detailed description of the process of developing the assessment domains that underpin the tool.

The Lowitja Institute is Australia’s national institute for Aboriginal and Torres Strait Islander health research. It is committed to working for the health and wellbeing of Australia’s First Peoples through research and knowledge exchange by providing funding to support a new generation of Indigenous health researchers. It began as a CRC for Aboriginal and Tropical Health (1997–2003), funded by the Australian Government CRC. A second CRC for Aboriginal Health followed (2003–2008). A third “bridging” CRC (2009–2014) supported a transition beyond CRCs to the Lowitja Institute. Like the predecessor CRCs, the Lowitja Institute seeks to bring Indigenous health research stakeholders – community-controlled organizations, service practitioners, policymakers, and researchers – into partnerships to ensure that research better addresses the needs and priorities of Indigenous people (5, 7).

Silburn et al. (7) evaluated the impact of a 5-year program of Lowitja Institute-funded research and development activities (2008–2013), acknowledging both successes and challenges. Successes included system-level improvements, such as the program of continuous quality improvement (CQI) in primary health care research, which, since 2015 has evolved into the Centre of Research Excellence in Integrated Quality Improvement. However, the evaluation did identify a significant gap in evidence linking research to impact (7). Silburn et al.’s review recommended three steps to improve research impact: (1) to ensure that data about research impact was collected and recorded in a systematic way; (2) to continue to collect data about research impact over time – even when initial projects have finished; and (3) to use impact indicators that Indigenous people value. These considerations led the Lowitja Institute to work collaboratively with the present authors to develop the research for impact tool designed to assist researchers and stakeholders to routinely plan and evaluate the impact of their research.

In developing the tool, the authors were guided by a broad research question: how can researchers improve the value of their research for society? Within this broad research question, there were two specific questions: what is the value of research? What are the challenges and opportunities involved in assessing the value of research in the context of Indigenous health? Throughout this paper, impact and value are used interchangeably to mean the benefits of research investment versus the costs.

## Materials and Methods

The methodology was a combination of literature reviews, workshops with researchers, both Indigenous and non-Indigenous, reflections by project team members and the Lowitja Institute partners – the executive who commissioned the tool and the Institute’s research support staff who worked closely with the authors to co-create the tool. Using participatory snowball techniques, the approach involved “plan–act–learn–plan–act cycles” (18–21), which are wholly consistent with the Lowitja Institute aims and Indigenous decolonized approaches to research (1). The tool development process occurred in three discrete phases, reflecting the plan–act–learn approach, with a fourth phase for the ongoing development and evaluation of a new toolkit.

### Phase 1 Plan

Scoping workshop and formation of project team: Author Komla Tsey, in his then role as a Lowitja Institute research program leader with particular interest in the impact agenda, was given oversight of the project. The original intention was to adapt the CRC Impact Tool and train the Lowitja Institute research applicants to undertake cost–benefit analysis of their research projects. The CRC Impact Tool is an input–output evaluation logic covering seven main domains: Inputs, Activities, Outputs, Usage, Impacts, Risk Analysis, and Net/Benefit (8).

The process began with University of Newcastle economists running a scoping workshop on research impact with 42 self-selected participants at Congress Lowitja 2014, the Institute’s biennial conference in Melbourne. Participants included researchers, policymakers, health service program coordinators, and managers interested in Indigenous health research. In small groups, they worked under guidance to apply a simplified CRC Impact Tool to real-life projects in which they were involved (22). Using the snowball approach, an open invitation was extended to participants interested in being involved in subsequent research impact workshops to contact the project team.

The CRC Impact Tool was a useful start, but workshop participants felt that in order to make the research impact assessment agenda meaningful and relevant, the main assessment domains of the tool should reflect Indigenous peoples’ research aspirations and interests. They pointed to numerous Indigenous research ethics guidelines and policy frameworks, such as the National Health and Medical Research Council (NHMRC) Values and Ethics Guidelines (23), Road Maps (24, 25), and Keeping Researchers on Track (26) as examples of evidence of what Indigenous people have been saying for many years that they wanted from research. Participants were particularly concerned about the risk of failing to adequately capture the so-called “intangible benefits,” such as cultural identity, relationships, control and ownership, and social and environmental wellbeing. Indigenous people value these benefits highly, but in the absence of reliable indicators and cost-efficient research design techniques, it was potentially difficult to capture the intangible benefits quantitatively in the existing CRC net/benefit framework.

Subsequent discussions of workshop feedback with the Lowitja Institute executive led to a decision to develop a new Lowitja Institute research impact tool explicitly to align with existing Indigenous research ethics and benefit frameworks. While the decision to develop a specific Lowitja Institute impact tool was exciting, it raised several challenges. A key challenge was how best to secure Indigenous ownership and participation in a project that was originally seen as the preserve of trained economists. Indigenous participants at the initial workshop, and others approached later, were interested in the research impact idea, but it was difficult and indeed unrealistic for people to sacrifice other commitments and priorities in order to become meaningfully involved in what was seen as a one-off short-term project.

Komla Tsey then invited Indigenous colleagues within his own research team and networks to consider using the short-term Lowitja Institute impact project, irrespective of any limitations it may have, as an opportunity to develop longer-term niche research interests in an emerging research space. Judging by feedback from the initial scoping workshop and subsequent discussions, research impact assessment was bound to become a high priority issue in the foreseeable future for Indigenous health research and for research more broadly. A Lowitja Institute research impact project team was constituted comprising the authors of this paper, who have had varying and relevant experience working in Indigenous health research (1).

### Phase 2 Act

Evidence search: To identify evidence regarding Indigenous peoples’ expectations and aspirations for research to inform the development of the research evaluation domains, searches of the websites of major Australian research bodies and relevant Indigenous organizations were conducted. These included the Australian Research Council (ARC), the NHMRC, the Australian Government CRC, the Lowitja Institute, the National Aboriginal Community Controlled Health Organisation (NACCHO), the Commonwealth Scientific and Industrial Organisation (CSIRO), the Australian Institute of Aboriginal and Torres Strait Islander Studies (AIATSIS), and the National Heart Foundation. A systematic search of the total publication output on Indigenous Australian health from 1995 to 2013 was conducted in order to generate baseline data regarding the extent to which researchers reported the impact of their research for Indigenous people as part of study results and the ways in which such impacts were framed.

Although a growing number of research impact tools have become available internationally and in Australia over the past 15 years, our evidence searches suggest that researchers are not necessarily using such tools to report the impact of their research, at least not in the peer-reviewed literature. For example, of 76 reviews of Indigenous health publications between 1992 and 2013 examined by Kinchin et al. (27), none focus on research impact or benefit assessment or related research evaluation, or include these issues in the review aims and objectives.

The NHMRC Indigenous research ethics and policy documents referred to above (23–26) were the most relevant documents found in terms of the explicit articulation of what Indigenous people expect of research. Despite minor differences, mainly deriving from the different contexts in which each of the documents originates, the key Indigenous research ethics principles found in the search have a common goal – to foster research integrity and maximize benefits for Indigenous people. Therefore, principles, such as Indigenous ownership and control, relationships based on respect and reciprocity, capacity building, benefit, and sustainability and transferability of benefits, underpin most of the documents.

Two of the most relevant Indigenous-specific research impact frameworks identified through the evidence searches were selected for closer examination (Table 1). The NHMRC Additional Criteria for Indigenous Health Research (28) was included because of its close alignment with the Lowitja Institute criteria. This was one of the rare instances where the criteria of a major funding body aligned with Indigenous community expectations of research, owing largely to the NHMRC’s process of extensive consultation with Indigenous community organizations and health leaders. These frameworks formed the basis for workshops on research evaluation domains and indicators (Phase 3 below) that were held for discrete research teams and groups in response to interest generated from the original scoping workshop.

**Table 1:** **Selected research impact frameworks**.

Impact framework	Domains of assessment
Lowitja Institute funding criteria (2013)	Aboriginal and Torres Strait Islander engagement Capacity development Collaboration and partnerships Outcomes for Aboriginal and Torres Strait Islander people
NHMRC Additional Criteria for Indigenous research (28)	Community engagement Benefit Sustainability and transferability Building capacity Priority and significance

### Phase 3 Learn

Impact assessment domains and indicators workshops. The NHMRC Additional Criteria and the Lowitja Institute funding criteria were systematically workshopped with Lowitja Institute research staff and with the authors’ research network. Two 1-day research impact workshops were conducted at James Cook University in Cairns for researchers from the authors’ network, followed by a 1-day workshop for Lowitja Institute research support staff in Melbourne (see Table 2). At both workshops, participants spent the day working through the NHMRC/Lowitja Institute funding criteria domain by domain as they identified, discussed, and debated the relevance, feasibility, and potential advantages and disadvantages of particular domains and indicators. Specifically, participants were asked to identify verifiable, relatively objective indicators that it was feasible for researchers to collect in order to assess research performance against the selected domains.

**Table 2:** **Indigenous research impact tool development workshops**.

Workshop location (date)	No. of participants	No. of indigenous participants (%)
James Cook University, Cairns (June 2014)	16	9 (56)
Lowitja Institute, Melbourne (June 2014)	11	2 (18)
Indigenous Health InfoNet, Perth (February 2015)	15	5 (33)
AIATSIS, Canberra (March 2015)	9	2 (22)
SAHMRI, Adelaide (April 2015)	13	7 (57)

### Phase 4 Ongoing Development and Evaluation of the Impact Tool

The learnings from phases 1–3 were combined to create a draft research impact tool. Subsequent workshops, designed to obtain feedback on the draft tool, were held for research networks at AIATSIS in Canberra, the Indigenous Health InfoNet at Edith Cowan University in Perth and at the South Australian Health & Medical Research Council (SAHMRI) in Adelaide (see Table 2). Using the participatory learning-by-doing approaches, the authors then worked closely with the Lowitja Institute staff, in regular teleconferences and face-to-face meetings, to negotiate and co-create, through numerous iterations, a practical research impact planning tool and supporting data templates.

Overall, the process of developing the impact tool and the underpinning assessment domains was necessarily organic and at times messy. Issues that arose were discussed continuously within the project team, during the workshops and with Lowitja Institute co-creators of the tool and lessons learnt were used to revise and modify both the focus of the project and numerous iterations of the tool development process. However, the findings are reported here in a broad, thematic approach to distil key learnings that emerged during the different stages and show how they influenced both the selection of the impact assessment domains and the methods proposed for working collaboratively with research users, funders, researchers, and other stakeholders to further develop and evaluate the tool.

## Results

The main findings from analysis of issues arising from the tool development process are presented under three main themes: the assessment domains underpinning the tool and how they were selected, the challenges of evaluating research and potential ways researchers might work round and, finally, an overview of the tool and feedback from workshop participants on its potential usefulness.

### The Assessment Domains and How They Were Selected

The stakeholder workshops revealed considerable consensus, interest, and support for a Lowitja Institute-led research impact agenda designed to improve the value of research for Indigenous people. It was agreed that as Lowitja Institute-funded research constituted only a small proportion of the total Indigenous health research output, a future research evaluation tool, if it is to have a meaningful impact on practice and, hence, improve research value, must have buy-in from the major research funding bodies, such as the NHMRC and the ARC. In other words, the Lowitja Institute research impact agenda should work in partnership with the major funders. Workshop participants also agreed that existing NHMRC funding criteria and other Indigenous ethics frameworks were useful domains that can guide researchers to plan and evaluate the impact of their research.

**Figure 1:**
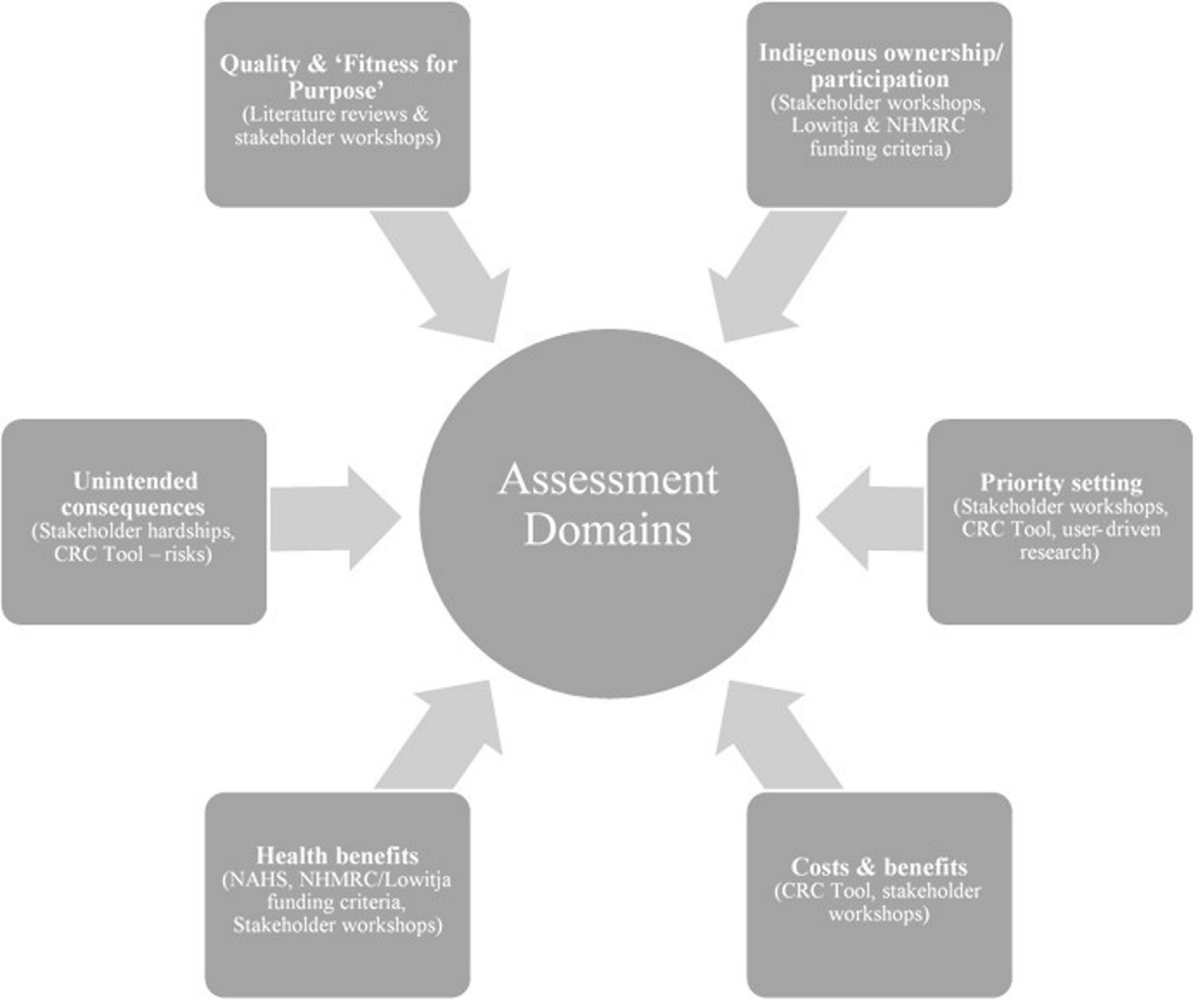
**Impact assessment domains**.

Figure 1 is an overview of the domains and how they were selected. Both the Indigenous ownership and the research priority setting domains derive from the strong consensus among workshop participants that these constitute the foundation from which to develop a truly Indigenous-led research, translation, and advocacy agenda. A key concern raised was that research relating to Indigenous people should be carried out on their terms, reflecting their rights of ownership and participation. In addition to the economic benefits of paid employment in research, less tangible but equally important benefits accrue from meaningful Indigenous ownership and participation including pride, a sense of achievement, and enhanced capacity and confidence. These intangible benefits are transferable to other settings.

Indigenous ownership and participation also relate to research priority setting. Setting the priorities for research can be motivated by a range of concerns. Participants found that the need for research can come from various sources, including Indigenous community organizations, health services, and other industry sectors. Other drivers of research include continuity of employment for the researchers, availability of new design techniques and data sources, intellectual curiosity, career aspirations, and the desire to be published.

Various anecdotes shared during stakeholder workshops and project team reflections make it clear that “research” in the conventional sense might not always be the most appropriate response. One such story told of a situation in a remote community where women and their babies were failing to turn up to their appointments with the visiting pediatric service. The issue was identified as a problem and a research team developed a $100,000 proposal to investigate the barriers to accessing the specialist service and strategies to improve that access. The community was approached and the community leaders asked the researchers to consult directly with the local health workers. This consultation revealed that the women were missing their appointments because the only available bus service did not run on the days that the service visited. In this case, a major but unnecessary research project – an outcome that workshop participants believed was common not only in Indigenous research, but research more broadly – was avoided by changing the timing of the pediatric visit to coincide with the availability of the bus. Clearly, non-research solutions, such as engagement, advocacy and quality improvement, should be considered as part of research priority setting.

Workshop participants argued that irrespective of the original motivation for research, anyone proposing or conducting research relating to Indigenous people must ensure that Indigenous people are given the opportunity to make informed decisions, from their point of view, regarding the relative priority of competing issues. This will require researchers and funders to invest considerable efforts in defining who the research users are, what information they need and how research can better support them in making smarter policy and practice decisions that help people achieve better health and wellbeing.

Research quality and “fitness for purpose” was included as a domain for assessment because of concerns regarding the poor state of Indigenous research, expressed at the workshops and backed up by extensive empirical literature (2, 29–34). Research to date has focused predominantly on description rather than intervention, and even the limited evaluations that have been conducted have been based on poor quality study design, contributing to the “sorry state” of Indigenous research (35). For research to achieve impact, the quality of the study design must be rigorous, credible, and trustworthy, as well as being appropriate or fit for the purpose of the study.

The health benefits domain draws on a combination of Lowitja Institute and NHMRC funding criteria, and on the National Aboriginal Health Strategy (NAHS) holistic definition of health as not just the absence of disease and illness, but also the social, emotional, and spiritual wellbeing of people and their relationships to their community and land (36). Therefore, health benefits are broadly defined to include benefits arising from the processes of carrying out the research, such as employment and capacity enhancement, and translating the knowledge into action, but it is left to researchers and their partners to identify benefits specific to their research contexts.

The costs and benefits domain draws on a combination of the CRC net/benefit framework (8, 22) and workshop participant support for evaluating research impact in terms of costs as well as of benefits. It was argued that an effective approach would be for research teams to begin by itemizing all research costs and benefits step by step, providing relevant quantitative and qualitative empirical data in support. This should generate valuable data on prospective costs and benefits to enable more informed economic evaluation at project levels and/or at broader research program levels.

### The “Wicked” Problem for Research Impact Evaluation

It was relatively easy to achieve consensus among workshop participants regarding the domains against which to assess research performance in the context of Indigenous research. The same cannot be said for how researchers might find indicators that are transparent, verifiable, and cost efficient to collect in order to assess these domains. Comments, such as “this is too hard”; “like searching for a needle in a hay stack”; “typical wicked problem”; “who has the right to define impact and benefit for Indigenous people”; and “community engagement, benefit, respect, reciprocity, capacity are all important but difficult to assess and rank objectively,” were common. One participant captured the sentiment at the workshops when she used the rhizome plant as a metaphor to illustrate the complex, ever-evolving, and uncertain nature of the pathways from research priority setting, through the conduct of research, and the application of knowledge to achieve impact. Citing Deleuze and Guattari (37), she explained: “Unlike trees or their roots, the rhizome connects any point to any other, and its traits are not necessarily linked to traits of the same nature; it brings into play very different regimes, signs and even non-sign states… It is comprised not of units but dimensions, or rather directions in motion …”

A qualitative narrative case study approach was often proposed as an alternative to the difficulty of quantifying benefits, such as engagement, capacity, respect, and reciprocity. In this qualitative approach, researchers provide impact statements, supported by evidence, which are then assessed against agreed criteria by panels made up of research users, funders, and other stakeholders. It was often also suggested that a meaningful evaluation of research would need to combine sets of objective measurable indicators with compelling evidence-informed impact narratives.

However, participants looking at the issues from a policy viewpoint cautioned against the use of qualitative narrative approaches without considerable testing in the field. They argue that these approaches are seen by policymakers to lack objectivity, are generally labor intensive and costly to undertake, and are, therefore, unappealing to governments and research funding agencies.

Overall, the message was that research evaluation is more than a technical exercise. It is a highly complex, serendipitous and potentially costly process, definitely a “wicked problem” for which there are no easy template solutions. In response, project participants advocated for a pragmatic approach where rather than trying to develop ready-made templates and leave it to researchers to gather data mechanistically, researchers and partners work together with the tool developers to apply the draft tool in real-life contexts and collectively learn from the process. Importantly, the approach needs to be guided by a particular understanding of the value of research, namely to create evidence and/or products to support society to make smarter decisions so as to improve the human condition. A practical way of approaching research impact is, therefore, to start with the decisions confronting decisions makers whether they are government policymakers, professional practitioners, or households and the extent to which the research supports them to make decisions that are smart and the knock-on consequences of such smart decisions. Smart decisions are decisions and choices that achieve particular outcomes with the least amount of resources and at the same time takes issues of equity and fairness into account.

### Overview of the Research for Impact Tool

**Figure 2:**
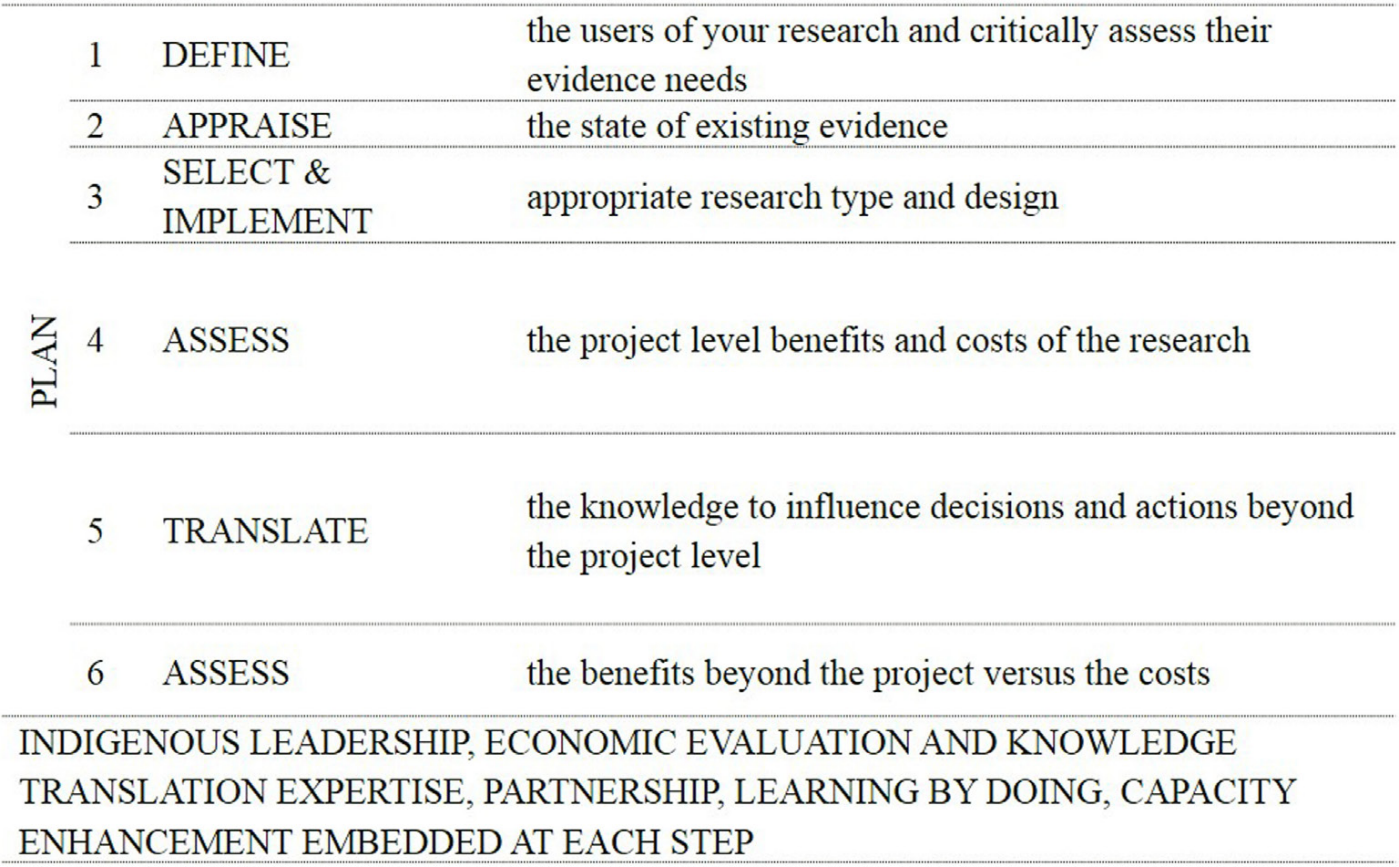
**Overview of the research for impact tool**.

Figure 2 is an overview of the Research for Impact Tool (Figure 2). The logic underpinning the impact process is based on the challenges and opportunities of assessing the impact of research discussed earlier and demonstrates how researchers can plan and track the impact of their research by having researchers first define their research users and their information needs clearly; then weigh up as objectively and honestly as possible the advantages and disadvantages of using existing versus additional information to inform users’ decisions; ensure that the selected research type and design quality are both rigorous and fit for purpose; develop project implementation and knowledge translation plans that are based on evidence as to what works; and use process and impact indicators to routinely monitor and evaluate the costs and benefits of the research. Embedded in the logic is the need for Indigenous leadership, participation, and capacity enhancement at each step in the process.

Feedback from follow-up workshops in Canberra, Perth, and Adelaide on an early version of the tool (see Table 2) was overwhelmingly positive. Of the 37 researchers who participated in these workshops, 24 (67%) returned the feedback sheet with comments. Sixty-three percent of these 24 participants have been working in the Indigenous research arena for more than 3 years. Thirty-eight percent of the 24 were Indigenous. Of the 24 who returned the feedback sheet, 58% agreed and 29% (87%; *n* = 21) strongly agreed that they found the tool useful for evaluating Indigenous research impact. Twelve percent were either neutral (8%; *n* = 2) or disagreed (4%; *n* = 1).

Specific comments, which were mainly positive, included “I think it incorporates the concepts of: transparency, principles, research into context, decision making tool, determining shared space, collaborative quality improvement”; “Makes the process explicit”; “No suggestions, however it is very interesting way to think more explicitly about broader-impact of research”; “I think this is a very good way to critically think about whether more research is needed in a certain field”; and “I look forward to seeing how this tool “plays out” in the future ☺.” Although excited about the prospect of a tool that can guide a systematic approach to assess research impact, participants also observed that more work needs to be done in order to objectively assess and compare performance across projects: “At the moment the tool is useful as a reflective tool, it makes you think and take everything into account. To make it relevant for policy decision making, e.g., what to fund and what not to fund, you need to calibrate and populate, with numerical values, so you can come up with a measure for each domain.” The next phase of the impact research is to work collaboratively with researchers to apply and evaluate the tool in real-life research contexts so the learnings can be used to refine the tool.

## Discussion

This paper set out to examine the process of developing a Research for Impact Tool in the contexts of general fiscal constraint, increased competition for funding, perennial concerns about the over-researching of Aboriginal and Torres Strait Islander issues without demonstrable benefits as well as conceptual and methodological difficulties of evaluating research impact. The aim was to highlight the challenges and opportunities involved in evaluating research impact to serve as resource for potential users of the research for impact tool and other interested researchers.

Although a growing number of research evaluation tools have become available internationally and in Australia, for example, the “Payback” tool and the CRC Impact Tool, our evidence searches, workshops, and project discussions suggest that researchers are not using such tools to report the impact of their research as part of research results (27). In other words, developing a research impact tool is one thing, having researchers use it to assess the performance of their research is another issue.

One reason the tools are not used more often is that most are not aligned with the funding criteria of the major research funding bodies. Consequently, researchers using such tools risked undermining the competiveness of their own funding proposals. Another reason the tools are not used is the perception that evaluating research impact is conceptually complex, difficult and potentially costly, a so-called “wicked problem” – something which the user guides to the existing tools have perhaps not emphasized as much as they should. From the point of view of research involving Indigenous people – historically oppressed minorities in their own country – the fact that existing tools are not aligned with the evidence of what they expect or want from research is another reason that they may not be appropriate. For example, they do not reflect that when research relates to Indigenous people, it needs to be carried out on their terms (1, 6, 14, 23, 25, 26, 38).

A key strength of the selected domains informing the Research for Impact Tool is that it brings together Indigenous expectations of research and major health research funding criteria from the NHMRC, providing the opportunity for all stakeholders – communities, funders, and researchers – to move in a common direction in evaluating research impact. Furthermore, framing research evaluation as a “wicked” rather than a mere “technical” problem provides the opportunity to tailor more appropriate participatory learning-by-doing approaches based on trust and reciprocity (18–21) to co-create mechanisms to evaluate the impact of research on health and wellbeing. As Rittel and Weber (39) remind us that there are no templates or ready-made answers when dealing with “wicked” problems because, as in the rhizome analogy, as soon as one problem is solved the very solution may trigger two or three other problems (39). The creation of collaborative spaces based on Aristotle’s concept of phronesis (40), for what Xiang (41) and other sustainability education advocates have called “deep learning” to occur between people of diverse backgrounds and interests, is vitally important in such circumstances. Deep learning is an adaptive, participatory, and transdisciplinary process of collective learning and exploration, promoting collaborative behavior, conflict management, trust among stakeholders, and better and more satisfying results (19, 21, 41).

The tool is grounded in two key concerns of research ethics. The first is the principles, values, and norms of Indigenous research ethics. These include the Lowitja Institute research principles, the NHMRC guidelines for funding and ethics, other best practice guides for Indigenous research (42, 43) and feedback from development workshops with researchers. These research principles and norms are designed to ensure that research is culturally sensitive and that benefits flow in ways valued by Indigenous people.

The second concern is understanding of the nature and value of research. The value of research, the authors believe, is to create evidence and/or products to help decision makers whether they are governments, businesses, service providers, or households to make smarter decisions that can have knock-on effects of improving the human condition. Research is, therefore, of limited value unless the evidence created is used to make smarter decisions for the betterment of society. From this point of view, defining the users of research and their information needs upfront will help researchers to better track and assess impact in terms of the extent to which their research supports users to make smarter policy and practice decisions. Focusing on the impact on users’ information needs does not limit any other impacts that may accrue from the research, but it helps to track progress toward the effects on users’ information needs as the primary impact measure while remaining sensitive to the range of other intended or unintended impacts and consequences. As the Lowitja Institute video cited at the beginning of this paper explained, “Good decisions flow from great research,” meaning to judge the value of research we must first know the quality of the decisions arising from the research.

This requires researchers to explicitly establish and justify, based on evidence, the users’ information needs and the intended use of new information produced by the proposed study and to honestly weigh up other ways to achieve similar or better outcomes than through the proposed research (44). In other words, researchers need to become better at information gap analysis, the explicit assessment of the state of existing evidence, the extent of information uncertainty, and the costs and benefits of generating new evidence to narrow the information gap. It is a key concern of the impact approach we are proposing to emphasize that the process of identifying users’ evidence needs and the best ways to meet such needs is research in its own right. Without explicit and transparent priority setting, we cannot improve the value of research and reduce waste. Priority-setting research is not something that researchers can do without dedicated funding. Considerable investment is required so that researchers can build the necessary relationships and partnerships to allow them to routinely identify potential evidence needs and undertake relevant evidence gap analysis. Researchers, research funders, and evidence users all need to be aware that until priority setting research is recognized and appropriately resourced, the value of research, namely the benefits of smarter policies and practices, will continue to be compromised before projects even begin.

We are aware that most of the world’s leading scientific and technological breakthroughs occur through serendipity, rather than as the result of targeted research. We contend, however, that targeted and serendipitous outcomes from research are not mutually exclusive. In fact, all research, whether vertical or horizontal or applied versus theoretical, do start with some potential benefit in mind even if not explicitly stated. Given the current environment of fiscal constraint and increased competition for funding and pressure to demonstrate a return on the investment, we believe that it is in the interests of all researchers, undertaking either applied or the so-called basic blue sky research, to become more alert to the information needs of potential users of their research, whether by chance or by design.

The domains informing the tool as they stand have some limitations. Participants in the tool develop workshops were mainly researchers, both Indigenous and non-Indigenous and, hence, do not necessarily reflect community and policy perspectives. Besides, the NHMRC and other Indigenous funding and ethical guidelines informing the tool were developed some 15 years ago and may need to be updated to reflect the changing needs of Indigenous Australians in 2016 and beyond. Recent guidelines (44) suggest that the earlier guides are still relevant today; nevertheless, changing policy and other influences on the research environment, such as the advent of technology and social connectivity via the internet and associated developments in telemedicine, genetic science, and personalized medicine that have emerged in that time should not be underestimated. Contemporary Indigenous Australians are likely to have different needs to those they had in 2000, and those needs are constantly evolving and must be assessed on an ongoing basis. In response to these and other challenges, Indigenous researchers within the project team took the lead in successfully winning a competitive ARC Discovery grant (Project ID: IN150100011) to undertake primary research exploring contemporary Indigenous constructions of research benefit.

To conclude, using the participatory learning-by-doing approaches, the authors are working closely with the Lowitja Institute staff to test and assess the tool in collaboration with Lowitja Institute-funded researchers and other research networks over the next 2 years (2016/17). The results of the ARC project to explore research benefit are informing the process. The baseline systematic literature search regarding the extent to which researchers reported the impact of their research as part of study results (27) will be repeated as part of broader monitoring and evaluation of the impact of the tool on research practice over time. The authors are also working closely with Lowitja Institute to engage with major funding bodies, such as the NHMRC, the ARC, and CRC, to ensure consistent alignment and approaches across research users, funders, and researchers in evaluating research impact into the future. The decision by the Australian Government to include ‘industry engagement’ and ‘impact’ as additions to the Excellence in Research for Australia (ERA) quality measures from 2018 makes the Research for Impact Tool a timely development.

The tool is designed in the context of Indigenous research but the basic idea that the way to plan, monitor, and evaluate research impact is to start upfront with the users’ information needs, the decisions confronting them and the extent to which research informs smarter decisions is equally applicable to research in other settings, both applied and theoretical. Beyond research, the tool can be adapted more broadly to prioritizing, monitoring, and evaluating policies, services, and programs.

## Author Contributions

KT conceptualized and drafted the paper; KL, IK, RB, JM, FW, YC-J, and AR made conceptual contributions and revised the paper.

## Conflict of Interest Statement

The authors declare that the research was conducted in the absence of any commercial or financial relationships that could be construed as a potential conflict of interest.
